# Comparative study of different kinesiology taping techniques to reduce postoperative morbidity after impacted mandibular third molar surgery

**DOI:** 10.4317/medoral.27400

**Published:** 2025-10-14

**Authors:** Dilek Menziletoglu, Arif Yigit Guler, Alparslan Esen

**Affiliations:** 1 Necmettin Erbakan University, Faculty of Dentistry, Oral and Maxillofacial Surgery, Konya, Turkey; 2 Medipol University, Faculty of Dentistry, Oral and Maxillofacial Surgery, Ankara, Turkey

## Abstract

**Background:**

The aim of the study was to compare and evaluate the effects of two different kinesio tape techniques on pain, swelling and trismus after mandibular impacted third molar surgery.

**Material and Methods:**

This study was designed as a controlled, randomized, prospective clinical trial. Mandibular third molars classified as Class II Position B according to Pell and Gregory classification and as mesioangular position according to Winter's classification were extracted. Patients divided into three groups. After third molar surgery, the classical kinesio tape technique (Technique A- kinesio tape which was divided into three equal segments extending from the cheek region to the clavicle) was applied to the patients in Group 1, while new technique (Technique B- both the classical KT extending from the cheek to the clavicle and an additional Y-shaped KT supporting the masseter muscle) was applied to patients in Group 2. No kinesio tape was placed to the patients in Group 3 during the postoperative period. Antibiotic, analgesic and a mouthwash were prescribed to all three groups. Pain was evaluated using with visial analog scale for 7 days postoperatively. Swelling was assessed on the 2nd and 7th postoperative days by taking measurements from five different points using a paper ruler. Maximum mouth opening was recorded using a caliper postoperatively on the second and seventh days.

**Results:**

Ninety patients (54 female, 36 male) were included in the study. Pain levels in Group 1 and Group 2 were statistically lower compared to Group 3. Statistical differences were found only on the 2nd and 3rd days between Group 1 and Group 2. The lowest pain level was observed in Group 2. Patients in Group 2 required the least amount of analgesics. Swelling in Group 1 and Group 2 was statistically lower than in Group 3. Statistically significant increases in mouth opening were observed in Group 1 and Group 2 compared to Group 3. No signs of infection were observed in the patients. The results of the quality of life scale indicated that patient satisfaction was highest in Group 2.

**Conclusions:**

Although kinesio tape applied with both different techniques was effective in reducing postoperative morbidity, Technique B was more effective.

** Key words:**Kinesio tape, third molar surgery, pain, trismus, swelling.

## Introduction

Surgical extraction of impacted third molars is a common practice in oral surgery [1]. In patients who have their impacted third molar extracted, trismus, pain, bleeding and edema may often be observed in the postoperative period. These complications affect patients' ability to interact with others and continue their daily routine activities, especially during the first three postoperative days [2]. These symptoms need to be managed professionally and reduced as much as possible. Many different methods have been tested over time to minimize postoperative complications associated with the extraction of impacted third molar teeth [3]. The most commonly preferred methods are low-dose laser therapy, non-steroidal anti-inflammatory drug use, cryotherapy, corticosteroid use, and manual lymphatic drainage [2,4-7]. Recently, kinesio tape (KT) application has also been recommended to minimize postoperative morbidity after the extraction of impacted third molar surgery. There are studies reporting that KT application has beneficial effects on pain, trismus and swelling [8-10]. KT, invented by Dr. Kase, is a form of elastic therapeutic tape. KT elevates the skin, and the inflammatory fluids in the tissues are directed to move from higher pressure areas to lower pressure areas under the influence of KT. As a result, the lymphatic congestion and the facial edema are reduced [11,12].

KT studies in dentistry generally consist of comparison of KT application with other different applications (drain, placebo, corticosteroid) [8,9,13]. To our knowledge, there is only one study comparing different application methods of KT in third molar surgery [1].

The purpose of this prospective clinical study was to examine the effects of KT applied with different techniques on trismus, swelling, and pain following mandibular impacted third molar extraction.

## Material and Methods

This prospective, controlled, randomized, clinical study was conducted on 90 patients aged 18-25 years who were referred to the Oral and Maxillofacial Surgery Clinic and who were indicated for extraction of mandibular impacted third molar teeth with mucosal and bone retention in the mesioangular position according to the Winter classification. Ethical approval fort he research was obtained from the Clinical Research Ethics Committee of Meram Medicine Faculty at Necmettin Erbakan University (08.03.2023-2023/1049). The study protocol was conducted in accordance with the Declaration of Helsinki. The purpose of the study, what the patient should do, and complications related to the surgery were explained to the patient, and an informed concent form was signed by patients who volunteered to participate in the study. The inclusion criteria for this study consist of patients without any systemic or psychiatric disease, aged between 18 and 25 years who are eligible for extraction under local anesthesia, and have impacted mandibular third molars retained in mucosa and bone, classified as mesioangular according to Winter's classification and as Class II, Position B according to the Pell and Gregory classification. Additionally, patients must not have pericoronitis or pathological lesion and should not have used antibiotics or analgesics for any reason within the past month. The exclusion criteria for this study include patients who smoke, those who use birth control pills, and individuals who are pregnant or breastfeeding. Additionally, patients with a known allergy to KT and those who had inflammatory reactions during the postoperative period were excluded from the study.

**Figure 1:**
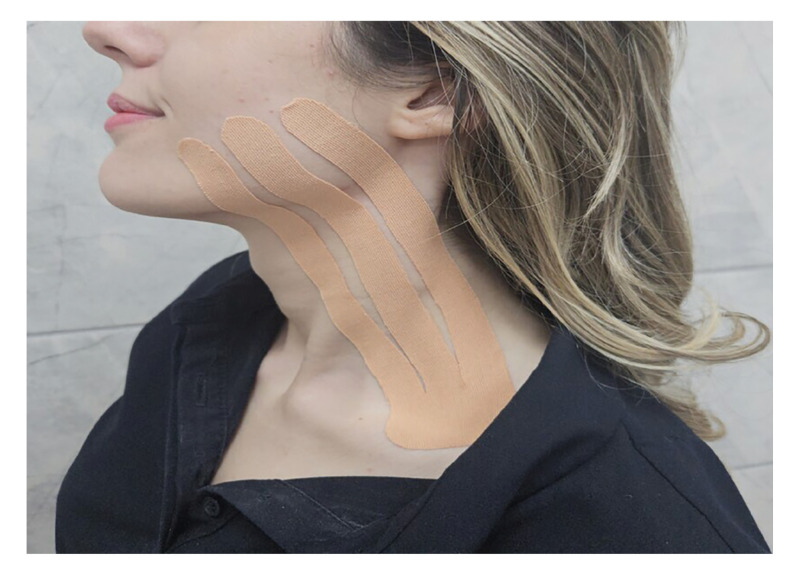
Application of kinesio tape using Technique A.

The study consisted of three groups (Group 1, Group 2, Group 3) and there were 30 patients in each group. The patients determined their groups themselves by drawing envelopes. Groups 1 and Group 2 were the research groups, and Group 3 was the control group. The same researcher performed all impacted tooth extractions under local anesthesia using the same technique in all groups, in accordance with the rules of asepsis and antisepsis. Inferior alveolar, buccal, and lingual nerve blockades were achieved with 2 ml of a local anesthetic containing 40 mg/ml articaine and 0.006 mg/ml epinephrine hydrocloride (Ultracaine DS, Sanofi Aventis, İstanbul, Türkiye) in three groups. A mucosal incision was made using a surgical scalpel, and the mucoperiosteal flap was raised. The osteotomy and crown sectioning were performed in all 90 extracted teeth using fissure and round burs with irrigation using sterile saline solution. The teeth were extracted using an elevator and dental forceps. The socket was curetted. The flap was repositioned and sutured using 3-0 silk suture. Patients in three groups were prescribed postoperative antibiotics (two times daily-Augmentin 1000mg Tablet, GlaxoSmith Kline, Istanbul, Turkey), analgesic (two times a daily- Arveles 25 mg Tablet, UFSA, Istanbul, Turkey) and mouthwash (three times daily for seven days- Kloroben, Drogsan, Ankara, Türkiye) Standard postoperative instructions were provided to the patients. The use of ice packs was discouraged due to the risk of masking swelling and pain. After tooth extraction, the skin of the patients in the research groups was cleaned, and KT was applied. In all KT applications, a skin-colored tape was preferred. The length of KT was adjusted individually for each patient. In Group 1, KT was applied using the classical technique described by Ristow *et al*. [14] (Technique A). Following the extraction of impacted third molars, KT was applied in three equal segments extending from the cheek region to the clavicle (Fig. 1).

**Figure 2:**
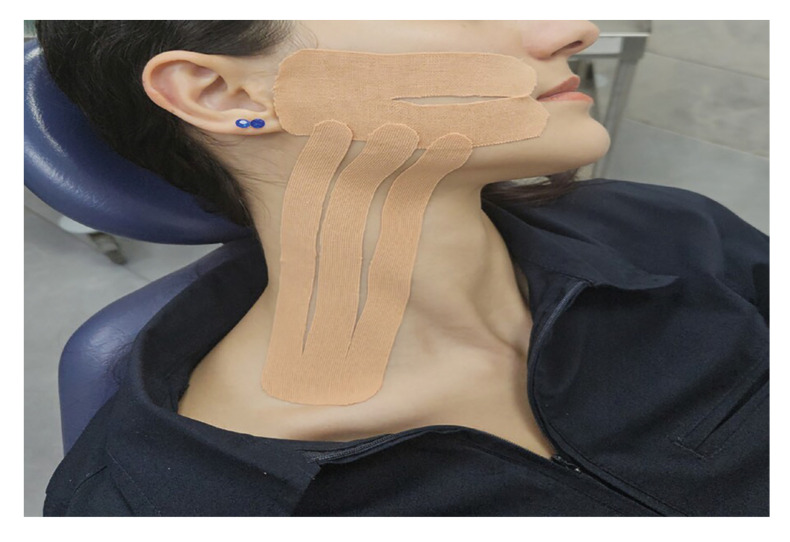
Application of kinesio tape using Technique B.

In Group 2, a novel KT technique (Technique B) defined by Gozluklu *et al*. [10] was applied. Technique B involved the application of both the classical KT extending from the cheek to the clavicle and an additional Y-shaped KT supporting the masseter muscle. In Technique B, the patient's mouth was gently opened, and the base of the Y-shaped tape was positioned at the temporomandibular joint region (Fig. 2). No KT was applied to the patients in Group 3 after their teeth were extracted. When the patients in Group 1 and Group 2 came to the clinic on the second postoperative day, KT was taken off. After swelling and trismus measurements were made, KT was reapplied. In both groups, KT were removed on the 5th day, and no further taping was performed. On the 7th postoperative day, the patients' sutures were removed.

-Evaluation of inflammatory symptoms and quality of life

Study variables were categorized into demographic, and outcome-related parameters. Demographic variables included age and gender. The primary outcome measures were pain, trismus, and edema, while quality of life scores were assessed as secondary outcomes. Baseline characteristics and demographic data were recorded preoperatively on the day of surgery. Inflammatory symptoms of operated sites were evaluated on the preoperative, postoperative second and seventh days.

The operation times were recorded for the three groups. The effects of KT techniques on postoperative pain, edema and trismus were evaluated. To assess edema, measurements were made just before the procedure and on the 2nd and 7th days after surgery, using a paper ruler to measure the distances between the tragus-pogonion, tragus-labial commissure, angulus mandible-lateral canthus and noted in millimeter (mm). Trismus was also recorded in mm by measuring the maximum interstitial distance (distance between the lower and upper central teeth) using a caliper just before the procedure and on the 2nd and 7th days after the surgery.

None of the patients reported pain prior to the procedure. Patients' pain complaints were assessed using a visual analog scale (VAS) every day during the one-week postoperative period. On the scale, a score of '0' represents no pain, while a score of '10' reflects the worst possible pain. Patients were asked to record the number of painkillers taken over the one-week period.

The global quality of life scale was used to assess the patients' quality of life after surgery [15]. Patients were asked to complete a global quality of life scale with scores ranging from 0 (poor) to 100 (excellent) on the 7th postoperative day to evaluate the one-week process (Table 1).

-Statistical Analysis

Statistical analyses were conducted using SigmaPlot 12.5 (Systat Software Inc., San Jose, CA, USA). Descriptive statistics, including minimum, maximum, mean values, and standard deviations, were calculated. Categorical data were analyzed with the chi-square test.

The Shapiro-Wilk test assessed the normality of distribution. For normally distributed data, One-Way ANOVA was applied, whereas the Kruskal-Wallis test was used for non-normally distributed data. Tukey and Bonferroni post-hoc tests were employed for multiple group comparisons. The significance level was set at *p* < 0.05

## Results

A total of 90 participants, 54 women (60%) and 36 men (40%), were enrolled in the study, with each group containing 30 patients. Gender distribution showed no statistically significant difference among the groups (*p* = 0.574). The patients had a mean age of 20.18 ± 2.24 years, and no significant age difference was found between the groups."(p>0.05)

The patients' pain values ​​were evaluated with VAS during the postoperative one-week period. A statistically significant difference was observed among the three groups. This difference continued for 7 days between the Group 3 and the research groups (Group 1, Group 2) (*p* <0.001). The pain levels between the research groups were similar throughout the week (Table 2).

Patients were asked to note the number of painkillers they used in the postoperative one-week period. The number of painkillers used by the patients was as follows according to the groups: 10,2±3,64 (Group 1), 8,13±3,69 (Group 2), 12,43±2,71 (Group 3). A significant difference was found between Group 1 and Group 3 (*p*=0.015), Group 2 and Group 3 (p0.001). The difference between Group 1 and Group 2 was not statistically significant (*p*=0.464).

To assess edema, measurements were made immediately before the procedure and on the 2nd and 7th days after surgery using a paper ruler to measure the distances between the tragus-pogonion, tragus-labial commissure, angulus-mandible-lateral canthus and noted in mm. In the measurements on both the 2nd and 7th days, a statistically significant difference was found between Group 1 and Group 3 (*p* <0.001) and between Group 2 and Group 3 (*p* <0.001). There was no statistical difference between Group 1 and Group 2 on the 2nd day (*p* = 0.424) and the 7th day (*p* = 0.979) (Table 3).

The maximum interstitial distance was measured in mm using a caliper before the procedure and on the 2nd and 7th days post-surgery to assess trismus. When comparing Group 1 and Group 3 on these days, Group 1 exhibited significantly greater mouth opening (*p*<0.05). Similarly, a significant difference was observed between Group 2 and Group 3 on both days (*p*<0.001). Although no statistical difference was found between Group 1 and Group 2 on either the 2nd or 7th day (*p* = 0.202; *p* = 0.315), the patients in Group 2 had slightly better mouth openings. When Group 1 and Group 3 were compared in the 2nd and 7th day measurements, the mouth opening was found to be statistically greater in Group 1 (*p* <0.05). Similarly, when Group 2 and Group 3 were compared in the 2nd and 7th day measurements, a significant difference was seen (*p* <0.001). There was no statistical difference between Group 1 and Group 2 in both the 2nd and 7th day measurements (*p* = 0.202; *p* = 0.315), but the mouth openings of the patients in Group 2 were found to be better (Table 4).

The operation times for the three groups were recorded. The operation time was 13.47 ± 2.64 minutes for Group 1, 13.53 ± 2.69 minutes for Group 2, and 12.40 ± 2.34 minutes for Group 3. No statistically significant differences were found among the three groups (*p* = 0.163).

On the 7th postoperative day, patients were asked to complete the global quality of life scale to assess their experience over the past week. Statistically significant differences were observed among the three groups (Group 1 vs. Group 2, *p* = 0.004; Group 1 vs. Group 3, *p* < 0.001; Group 2 vs. Group 3, *p* < 0.001).

The quality of life scores were 73.33 ± 6.34 in Group 1, 79.33 ± 6.39 in Group 2, and 59.33 ± 8.07 in Group 3. Patient satisfaction was found to be the highest in Group 2. None of the patients in the 3 groups had poor quality of life. While 60% of the patients in the control group had moderate or above quality of life, all patients in Groups 1 and 2 had moderate or above quality of life.

## Discussion

KT is designed to have a lifting effect on the skin on which it is applied and to allow air circulation between the skin and the external environment. KT technique is based on three basic concepts. These are area, cooling, and movement. Since painful and inflamed muscles swell due to edema, the area in which they are located narrows. When KT is applied, the skin is lifted, and since the subcutaneous interstitial space and skin are increased, circulation and movement also increase. Increased movement and circulation reduce inflammation in that area and the area begins to cool. In this way, the aim is to reduce pain, increase performance, reeducate the neuromuscular system, prevent injury, and accelerate circulation and tissue healing [16]. It has also been determined that KT reduces the pressure on nociceptors in the area where it is applied, thus reducing pain. The movements of the face stretch the KT, thus adjusting the KT length to the length of the skin, stimulating the skin and distracting the patients’ attention from perception and pain [17]. KT is commonly applied in orthopedics, sports medicine, physical therapy and rehabilitation departments. This technique has recently become popular in oral surgery. There are very few scientific publications examining the effectiveness of KT in oral and maxillofacial surgery. These publications evaluated the effects of KT on orthognathic surgery, maxillofacial trauma, and impacted tooth surgery [3,10,14,18]. In our study, we applied KT in two different techniques after mandibular impacted third molar extraction and evaluated pain, edema, and trismus. Upon examining the pain outcomes, a statistically significant difference was observed across all three groups. Pain levels in Group 3 were significantly higher than those in Group 1 and Group 2 over the week. No significant difference was found between Group 1 and Group 2 on the first two days; however, a difference emerged on days 3 and 4. In the following days, there was no observed difference in pain between these two groups. Pain levels were lower in Group 2, where KT was applied using Technique B. We concluded that Technique B was more effective in reducing pain compared to Technique A. All these results indicate that KT applied to Group 2 had a significant effect in lowering VAS scores. We believe that this effect is due to the tape placed in the masseter region, which supports the masseter muscle and alleviates pressure on the nociceptors, resulting in further pain reduction.

Yurttutan *et al*. [19] included 30 patients who had their impacted teeth extracted, and applied KT to one group and no KT to the other group. They investigated whether KT had any effect on painkiller use in the postoperative period and found that patients used less analgesics in KT group. Tatli *et al*. [9] extracted the mandibular impacted third molars of 60 patients and divided the patients into three groups. They placed KT in Group 1, placebo KT in Group 2 and perform no KT in Group 3. It was found that the number of painkillers used by the patients in Group 1 was less than the other 2 groups. In our study, the number of painkillers consumed in Group 1 and Group 2 was less than Group 3. Among the three groups, it was determined that the patients in Group 2 consumed the least number of painkillers. The pain values ​​of patients in Group 2 were also lower on the VAS scale that the patients filled out. The less pain also reduced the number of painkillers used.

One of the postoperative complications of third molar surgery is swelling. Swelling reaches its maximum level within 24-48 hours and decreases within 5-7 days [10,20]. In recent years, some studies have used KT application to reduce swelling after impacted third molar extractions. It is a complementary treatment method that does not have any side effects and tries to affect the swelling with completely mechanical methods [21]. Patil *et al*. [3] included 15 patients have bilateral mandibular third molar in a study where they randomly applied KT tape to one side of the patients' faces after extraction, and did not apply KT tape to the other side. When they evaluated the effect of KT on swelling, they stated that KT was beneficial in reducing swelling. Tatlı *et al*. [9] evaluated whether KT application and placebo KT application had an effect on swelling after impacted third molar extraction and reported that KT application was effective, but placebo KT application was not effective. KT had an effective outcome in reducing facial swelling in orthognatic surgery [18] and the treatment of mandibular fracture [21]. Gozluklu *et al*. [10] compared the classical KT technique with KT technique applied to the masseter region in addition to the classical KT application to evaluate postoperative swelling in impacted third molar surgery. They found less swelling values ​​in the technique with additional bandaging. When we examined the swelling values ​​in our study, the amount of swelling in Group 1 and Group 2 was similar on both the 2nd and 7th days, but the swelling values ​​in Group 2 were less. When the swelling values ​​were compared with Group 3 and the research groups (Group 1 and Group 2), a significant difference was found between them. The highest swelling values ​​among the three groups were in Group 3. Technique B was found to be more effective in reducing edema compared to Technique A. We believe that this effect is due to the increased space between the skin and subcutaneous connective tissue created by Technique B, which accelerates blood and lymphatic drainage, leading to a faster reduction of edema.

Postoperative mouth opening is directly related to swelling and pain [3]. As the patients' pain and facial swelling decrease, trismus also decreases. Gozluklu *et al*. [10] reported that 2 different KT band techniques applied to the face after impacted third molar extraction had a positive effect on trismus and that mouth opening returned to normal in a shorter time after KT application. Contrary to this study, Tozzi *et al*. [18] reported that KT applied after orthognathic surgery had no effect on trismus. A noTable difference was observed in our study when mouth opening was evaluated among the three groups. Mouth restriction was seen the most in Group 3. No significant differences was showedbetween Group 1 and Group 2 in the 2nd and 7th day measurements. Trismus findings were parallel to edema and pain values. We believe that the rapid normalization of mouth opening is due to KT applied with Technique B, which not only supports the masseter muscle but also contributes more effectively to drainage. By reducing swelling and relieving skin tension, this technique facilitates faster recovery of mouth opening.

Quality of life is a multidimensional concept that encompasses various elements such as individuals' health status, functional capacity and general life satisfaction. Measurements to assess quality of life are usually carried out through questionnaires or scales. Postoperative quality of life assesses the impact of surgical procedures on daily functional activities and the level of postoperative pain. Previous research has demonstrated a decline in quality of life following both third molar surgeries and routine tooth extractions [22,23]. Patil *et al*. [3] reported that KT band applied after impacted third molar surgery helped to regain quality of life. In our study, KT methods applied positively affected the quality of life of the patients. The highest patient satisfaction was found in Group 2.

A limitation of our study could be the absence of a placebo taping group. We believe that in future studies on this subject, it would be beneficial to compare KT techniques with placebo KT techniques in a larger population by increasing the number of patients.

## Conclusions

Kinesio tape application is a simple and beneficial method that can be preferred to minimize postoperative morbidity following impacted third molar extraction. Both KT techniques were effective in reducing swelling, trismus, and pain but Technique B yielded better results in our study. KT application helps patients have a more comforTable postoperative period, consume fewer painkillers, and contributes to an improvement in their quality of life.

## Figures and Tables

**Table 1:** Global quality of life scale.

Global Quality of Life Scale
100 - Excellent quality of life
95 - Near-excellent quality of life
90
85 - Very good quality of life
80
75
70
65
60- Moderate quality of life
55
50
45
40 - Slightly poor quality of life
35
30 - Poor quality of life
25
20
15- Very poor quality of life
10
5- Extremely poor quality of life
0- No quality of life

**Table 2:** Comparison of pain at different intervals among the groups with VAS values.

Period of evaluation	Group 1 (Median / Mean±SD)	Group 2 (Median / Mean±SD)	Group 3 (Median / Mean±SD)	p
Day 1	6/6.13±1.47	6/5.2±1.63	8/ 7.73±1.46	p =0.241 γ p =0.005 α p <0.001 *
Day 2	6/4.93±2.12	6/4.27±1.26	6/6.57±1.77	p =0.297 γ p =0.014 α p <0.001*
Day 3	5/3.4±1.85	4/2.93±1.64	6/5.2±1.45	p =0.003 γ p =0.153 α p <0.001*
Day 4	4/2.93±1.46	2/2±1.29	4/4.27±1.64	p =0.05 γ p =0.045 α p <0.001*
Day 5	2/2.13±1.57	2/1.33±1.42	4/3.2±1.45	p =0.212 γ p =0.067 α p <0.001*
Day 6	0/1.07±1.26	0/0.53±1.17	2/2.53±1.74	p =0.431 γ p =0.010 α p <0.001*
Day 7	0/0.53±1.17	0/.4±0.81	2/1.47±1.38	p =0.894 γ p =0.025 α p =0.016*

( γ Group 1versus Group 2; α Group 1 versus Group 3; *Group 2 versus Group 3).

**Table 3:** Comparison of swelling measurements by group.

Period of evaluation	Swelling measurements			p
	Group 1 (Median / Mean±SD)	Group 2 (Median / Mean±SD)	Group 3 (Median / Mean±SD)	
Preoperative	119.67/120.36±8.72	120/121.58±8.88	115/ 117.20±6.10	0.246
Day 2	123.33/ 126.51±8.18	125/126.96±9.66	123/125.07±6.39	p = 0.424 ^γ^ p <0.001 α p <0.001*
Day 7	120/122.82±8.8	121/123.96±8.93	118/120.69±4.87	p = 0.979 ^γ^ p <0.001 α p <0.001*

^γ^ Group 1versus Group 2; ^α^ Group 1 versus Group 3; *Group 2 versus Group 3.

**Table 4:** Comparison of mouth opening measurements.

Period of evaluation	Mouth opening			p
	Group 1 (Median / Mean±SD)	Group 2 (Median / Mean±SD)	Group 3 (Median / Mean±SD)	
Preoperative	43/42.07±4.76	41/43.27±3.76	43/44.07±4.99	0.853
Day 2	30/31.33±6.76	30/31.8±4.39	20/23.8±8.53	p = 0.202 γ p <0.05 α p <0.001*
Day 7	40/39.88±2.94	40/38.13±3.54	32/32.67±4.06	p = 0.315 γ p <0.05 α p <0.001 *

γ Group 1versus Group 2; α Group 1 versus Group 3; *Group 2 versus Group 3.

## References

[1] da Rocha Heras ACT, de Oliveira DMS, Guskuma MH, de Araújo MC, Fernandes KBP, da Silva Junior RA (2020 Feb). Kinesio taping use to reduce pain and edema after third molar extraction surgery: A randomized controlled split-mouth study. J Craniomaxillofac Surg.

[2] Grossi GB, Maiorana C, Garramone RA, Borgonovo A, Creminelli L, Santoro F (2007 May). Assessing postoperative discomfort after third molar surgery: a prospective study. J Oral Maxillofac Surg.

[3] Patil S, Rajanikanth K, Bhola N (2023 Dec). Efficacy of Kinesio taping in post operative sequalae after surgical removal of mandibular third molars: a split mouth randomized control study. BMC Oral Health.

[4] Benetello V, Sakamoto FC, Giglio FPM, Sakai VT, Calvo AM, Modena KCS (2007 Aug). The selective and non-selective cyclooxygenase inhibitors valdecoxib and piroxicam induce the same postoperative analgesia and control of trismus and swelling after lower third molar removal. Braz J Med Biol Res.

[5] Canellas JVDS, Ritto FG, Tiwana P (2022 Oct). Comparative efficacy and safety of different corticosteroids to reduce inflammatory complication safter mandibular third molar surgery: A systematic review and network meta-analysis. Br J Oral Maxillofac Surg.

[6] Hamid MA (2017 Jul). Low-level laser therapy on postoperative pain after mandibular third molar surgery. Ann Maxillofac Surg.

[7] Laureano Filho JR, De Oliveira e Silva ED, Camargo IB, Gouveia FMV (2005 Jun). The influence of cryotherapy on reduction of swelling, pain and trismus after third-molar extraction: a preliminary study. J Am Dent Assoc.

[8] Genc A, Cakarer S, Yalcin BK, Kilic BB, Isler SC, Keskin C (2019 Jan). A comparative study of surgical drain placement and the use of kinesiologic tape to reduce postoperative morbidity after third molar surgery. Clin Oral Investig.

[9] Tatli U, Benlidayi IC, Salimov F, Guzel R (2020 Jul). Effectiveness of kinesio taping on postoperative morbidity after impacted mandibular third molar surgery: a prospective, randomized, placebo-controlled clinical study. J Appl Oral Sci.

[10] Gozluklu O, Ulu M, Gozluklu HO, Yilmaz N (2020 May). Comparison of different kinesio taping techniques after third molar surgery. J Oral Maxillofac Surg.

[11] Ouyang J, Chang K, Hsu W, Cho Y, Liou T, Lin Y (2018 Jan). Nonelastic taping, but not elastic taping, provides benefits for patients with knee osteoarthritis: systemic review and meta-analysis. Clin Rehabil.

[12] Melese H, Alamer A, HailuTemesgen M, Nigussie F (2020 May). Effectiveness of kinesio taping on the management of knee osteoarthritis: a systematic review of randomized controlled trials. J Pain Res.

[13] Erdil A, Akbulut N, Altan A, Demirsoy MS (2021 Apr). Comparison of the effect of therapeutic elastic bandage, submucosal dexamethasone, or dexketoprofen trometamol on inflammatory symptoms and quality of life following third molar surgery: a randomized clinical trial. Clin Oral Investig.

[14] Ristow O, Hohlweg-Majert B, Stürzenbaum SR, Kehl V, Koerdt S, Hahnefeld L (2014 May). Therapeutic elastic tape reduces morbidity after wisdom teeth removal: a clinical trial. Clin Oral Investig.

[15] Hyland ME, Sodergren SC (1996 Oct). Development of a new type of global quality of life scale, and comparison of performance and preference for 12 global scales. Qual Life Res.

[16] Cools AM, Witvrouw EE, Danneels LA, Cambier DC (2002 Aug). Does taping influence electromyographic muscle activity in the scapular rotators in healthy shoulders?. Man Ther.

[17] Gonzalez-Iglesias J, Fernandez-de-Las-Penas C, Cleland JA, Huijbregts P, Gutiérrez-Vega MDR (2009 Jul). Short-term effects of cervical kinesio taping on pain and cervical range of motion in patients with acute whiplash injury: A randomized clinical trial. J Orthop Sports Phys Ther.

[18] Tozzi U, Santagata M, Sellitto A, Tartaro GP (2016 Mar). Influence of kinesiologic tape on post-operative swelling after orthognathic surgery. J Maxillofac Oral Surg.

[19] Yurttutan ME, Sancak KT (2020 Sep). The effect of kinesio taping with the web strip technique on pain, edema, and trismus after impacted mandibular third molar surgery. Niger J Clin Pract.

[20] Slomka B, Rongies W, Ruszczuk P, Sierdzinski J, Saganowska D, Zdunski S (2018 Jul). Short-term effect of kinesiology taping on temperature distribution at the site of application. Res Sports Med Print.

[21] Ristow O, Hohlweg-Majert B, Kehl V, Koerdt S, Hahnefeld L, Pautke C (2013 Aug). Does elastic therapeutic tape reduce postoperative swelling, pain, and trismus after open reduction and internal fixation of mandibular fractures?. J Oral Maxillofac Surg.

[22] Adeyemo WL, Taiwo OA, Oderinu OH, Adeyemi MF, Ladeinde AL, Ogunlewe MO (2012 Oct). Oral health-related quality of life following non-surgical (routine) tooth extraction: A pilot study. Contemp Clin Dent.

[23] Bradshaw S, Faulk J, Blakey GH, Phillips C, Phero JA, White RP (2012 Nov). Quality of life outcomes after third molar removal in subjects with minor symptoms of pericoronitis. J Oral Maxillofac Surg.

